# New Proteins Contributing to Immune Cell Infiltration and Pannus Formation of Synovial Membrane from Arthritis Diseases

**DOI:** 10.3390/ijms23010434

**Published:** 2021-12-31

**Authors:** Dominique de Seny, Dominique Baiwir, Elettra Bianchi, Gaël Cobraiville, Céline Deroyer, Christophe Poulet, Olivier Malaise, Geneviève Paulissen, Marie-Joëlle Kaiser, Jean-Philippe Hauzeur, Gabriel Mazzucchelli, Philippe Delvenne, Michel Malaise

**Affiliations:** 1 Laboratory and Service of Rheumatology, GIGA Research, Centre Hospitalier Universitaire de Liège, University of Liège, 4000 Liège, Belgium; Gael.Cobraiville@chuliege.be (G.C.); celine.deroyer@chuliege.be (C.D.); christophe.poulet@chuliege.be (C.P.); Olivier.Malaise@chuliege.be (O.M.); Genevieve.Paulissen@chuliege.be (G.P.); mjkaiser@chuliege.be (M.-J.K.); jean-philippe@hauzeur.org (J.-P.H.); michel.malaise@chuliege.be (M.M.); 2 GIGA Proteomics Facility, University of Liège, 4000 Liège, Belgium; D.Baiwir@uliege.be (D.B.); p.delvenne@chuliege.be (P.D.); 3 Department of Pathology, GIGA Research, Centre Hospitalier Universitaire de Liège, University of Liège, 4000 Liège, Belgium; ebianchi@chuliege.be; 4 Mass Spectrometry Laboratory, MolSys Research Unit, University of Liège, 4000 Liège, Belgium; Gabriel.Mazzucchelli@uliege.be

**Keywords:** proteomics, synovial membrane, inflammation, LAP3, DNAJB11, MANF, LCP1, CTSZ, PTPRC, EML4

## Abstract

An inflamed synovial membrane plays a major role in joint destruction and is characterized by immune cells infiltration and fibroblast proliferation. This proteomic study considers the inflammatory process at the molecular level by analyzing synovial biopsies presenting a histological inflammatory continuum throughout different arthritis joint diseases. Knee synovial biopsies were obtained from osteoarthritis (OA; n = 9), chronic pyrophosphate arthropathy (CPPA; n = 7) or rheumatoid arthritis (RA; n = 8) patients. The histological inflammatory score was determined using a semi-quantitative scale based on synovial hyperplasia, lymphocytes, plasmocytes, neutrophils and macrophages infiltration. Proteomic analysis was performed by liquid chromatography-mass spectrometry (LC-MS/MS). Differentially expressed proteins were confirmed by immunohistochemistry. Out of the 1871 proteins identified and quantified by LC-MS/MS, 10 proteins (LAP3, MANF, LCP1, CTSZ, PTPRC, DNAJB11, EML4, SCARA5, EIF3K, C1orf123) were differentially expressed in the synovial membrane of at least one of the three disease groups (RA, OA and CPPA). Significant increased expression of the seven first proteins was detected in RA and correlated to the histological inflammatory score. Proteomics is therefore a powerful tool that provides a molecular pattern to the classical histology usually applied for synovitis characterization. Except for LCP1, CTSZ and PTPRC, all proteins have never been described in human synovitis.

## 1. Introduction

The synovial membrane is a thin connective tissue that separates the joint capsule from the joint cavity. It contributes to cartilage nutrition and lubrication by maintaining synovial fluid volume and composition. It comprises two layers: the intima (the inner lining layer in contact with synovial fluid in the joint cavity) and subintima (the outer sublining layer in contact with the joint capsule). The intima consists of 1–2 cells thickness, including fibroblasts and resident macrophages, while the subintima is relatively acellular, containing blood and lymphatic vessels, fibroblasts and few infiltrating cells in a collagenous extracellular matrix [1]. The synovial membrane is known for playing a major role in the inflammatory joint diseases such as rheumatoid arthritis (RA) but also to a less extent in osteoarthritis (OA) [2].

Increasing numbers of infiltrated immune cells (macrophages, dendritic cells, mast cells, natural killer cells, innate lymphoid cells but also B and T lymphocytes, plasma cells) and fibroblasts contribute to the synovial inflammation and pannus formation. It drives joint inflammation and thereby the destruction of the articular cartilage and bone. Single-cell RNAseq studies have shown that fibroblasts and macrophages from patients with active RA and OA are heterogenous [3,4,5,6]. Culemann et al. recently identified CX3CR1+ lining-layer macrophages that form a protective epithelial-like barrier in physiological condition [7]. This tight-junction-mediated shield protects intra-articular structures and thereby controls the onset of inflammation [7]. It can be disrupted during experimental arthritis, as also observed in patients with RA [7]. RA is a chronic inflammatory joint disease of autoimmune nature for which the synovium is a central player [8,9]. It is characterized by synovial inflammation based on innate and adaptative cell infiltration. Macrophages, which are probably differentiated from blood-derived monocytes, drive T-cell infiltration via antigen presentation [10]. This action can trigger B-cell infiltration and enhance the production of immunoglobulins and rheumatoid factor. Macrophages are the main producers of TNF-α. Secretion of cytokines and chemokines perpetuates the inflammatory response by recruiting additional innate immune cells, such as monocytes and neutrophils, also by inducing T-cell differentiation. Distinct fibroblast subsets in the synovial sublining can also play a critical role for both joint damage and inflammation [3]. They exhibit migratory activity and local proliferation, and release a high level of metalloproteinases, cytokines and chemokines [11]. Inflammation of the synovial membrane is also observed in OA, even in the early stage of the disease [2,12,13,14]. OA is the most prevalent joint disease worldwide. It is mainly characterized by cartilage degradation, osteophytes formation and subchondral bone sclerosis. OA was long considered as a degenerative disease, but it is now well described as a pathology presenting local inflammatory features [2,13,14]. Debris resulting from cartilage degradation such as hyaluronic acid fragments, are recognized as DAMPs (damage-associated molecular pattern) by TLRs (Toll-like receptors) present on the surface of synovial cells. Activation of these TLRs in turn leads to the expression of transcription factors, including NF-κB, responsible for the production of pro-inflammatory cytokines (IL-1, IL-6, TNF-α) and chemokines (IL-8) [2]. TLRs activation and cytokines/chemokines production become therefore major inducers of immune cells recruitment [14]. Further, proliferation of endothelial cells contributes to synovial membrane neovascularization strengthening the influx of the immune cells [13]. OA and RA pathologies are distinct for many physiopathological properties, but they also share some common features, including synovitis and pannus formation. Presence of calcium pyrophosphate crystal in the joint tissues of chronic pyrophosphate arthropathy (CPPA) patients induce synovitis similarly to OA and RA. In a previous study, we observed that levels of endoplasmic reticulum (ER) stress proteins, mostly chaperones and co-chaperones, were increased in synovitis of OA, CPPA and RA patients and that their expression levels were correlated with the histological inflammatory scoring based on the immune cell infiltration and hyperplasia [15]. In this proteomic study performed on the same patients as previously published [15], we further focus on proteins differentially expressed in the highly inflamed synovitis of RA patients compared to moderately inflamed CPPA and OA synovitis to better understand the inflammatory process that occurs in the tissue at the molecular level. Synovitis is a common clinical presentation for these three pathologies but various pathogenic pathways may also occur alone or in combination. We therefore compared protein levels obtained by mass spectrometry to protein quantified and localized by immunohistochemistry on synovial tissue provided from the same patients.

## 2. Results

### 2.1. Proteomic Analysis

**Figure 1:**
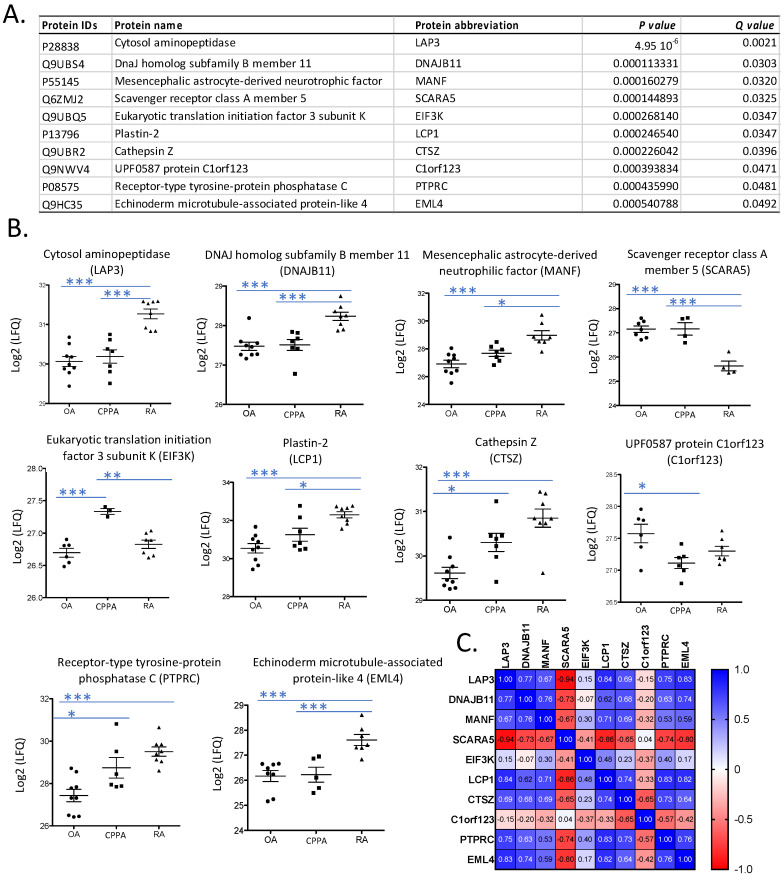
Proteomic analysis by mass spectrometry of synovial membrane from OA, CPPA and RA patients. (**A**) Protein expressions significantly modulated in OA, CPPA or RA synovial biopsies using the multiple sample test with 1400 permutation-based FDR for statistical analysis. (**B**) Representation of protein expressions obtained by mass spectrometry (Log2 (LFQ)) for the three pathologies. One-way ANOVA test with a post hoc test of Tukey was applied on logarithmic values: * *P* < 0.05, ** *P* < 0.01 and *** *P* < 0.001. (**C**) Correlation coefficients between the 10 highlighted biomarkers calculated according to the parametric Pearson test. OA, osteoarthritis; CPPA, chronic pyrophosphate arthropathy; RA, rheumatoid arthritis. Gene name was used to abbreviate protein name.

Synovial biopsies obtained from patients with knee OA (n = 9), CPPA (n = 7) and RA patients (n = 8) were provided from treatment-naïve patients as previously described [15]. A proteomic analysis was then performed on the 24 biopsies by 2D-nano-UPLC-ESI-Q-Orbitrap for protein identification and quantification [15]. In total, 1871 proteins were identified and selected for statistical analysis according to their quantification in at least seven biopsies of at least one of the three disease groups. The 1871 proteins were then submitted to the multiple sample test of 1400 permutation-based FDR. Ten proteins were significantly modulated among the three groups as presented in Figure 1A. Gene name was used to abbreviate protein name for fluent reading.

Seven protein levels were significantly increased in RA compared to OA and/or CPPA (Figure 1B): cytosol aminopeptidase (LAP3), DnaJ homolog subfamily B member 11 (DNAJB11), mesencephalic astrocyte-derived neurotrophic factor (MANF), plastin-2 (LCP1), cathepsin Z (CTSZ), receptor-type tyrosine-protein phosphatase C (PTPRC) and echinoderm microtubule-associated protein-like 4 (EML4). LAP3, DNAJB11, LCP1 and CTSZ were detected in the 24 biopsies. MANF was expressed in 23 biopsies (9/9 OA, 7/7 CPPA and 7/8 (87%) RA), PTPRC in 23 biopsies (9/9 OA, 6/7 (86%) CPPA and 8/8 RA), EML4 in 20 biopsies (8/9 (89%) OA, 5/7 (71%) CPPA and 7/8 (87%) RA). Scavenger receptor class A member 5 (SCARA5) protein levels were significantly decreased in RA compared to OA and CPPA and detected in 15 biopsies (7/9 (78%) OA, 4/7 (57%) CPPA and only 4/8 (50%) RA). Eukaryotic translation initiation factor 3 subunit K (EIF3K) protein levels were significantly increased in CPPA compared to the other groups and detected in 16 biopsies: 6/9 (67%) OA, 3/7 (43%) CPPA and 7/8 (87%) RA. Lastly, UPF0587 protein C1orf123 (C1orf123) levels were significantly increased in OA compared to the other groups and detected in 19 biopsies (6/9 (67%) OA, 6/7 (86%) CPPA and 7/8 (87%) RA).

Correlations between the ten selected proteins are shown in Figure 1C. In summary, SCARA5 is negatively correlated with all proteins, except C1orf123. EIFK3 is not correlated with any protein, except negatively with SCARA5 and positively with LCP1. C1orf123 is not correlated with any protein, except negatively with CTSZ and PTPRC. All other proteins are positively correlated to each other.

Lastly, we analyzed the correlation between these 10 proteins expression and the histological inflammatory score. This histological inflammatory score, including synovial hyperplasia, lymphocyte, plasmocyte, PMN and macrophage infiltration, was previously calculated for each biopsy and illustrated in our previous proteomic publication [15]. Briefly, this score was in the range of 3 to 8 for OA, 5 to 13 for CPPA and 12 to 17 for RA illustrating an inflammatory continuum throughout the 24 biopsies with an overlap between the three pathologies. All protein levels that were upregulated in RA, were significantly and positively correlated with the histological inflammatory score (HIS) (Table 1): LAP3 (r = 0.77, *P* < 0.0001), DNAJB11 (r = 0.77, *P* < 0.0001), MANF (r = 0.79, *P* < 0.0001), LCP1 (r = 0.74, *P* < 0.0001), CTSZ (r = 0.69, *P* = 0.0002), PTPRC (r = 0.68, *P* = 0.0004) and EML4 (r = 0.78, *P* = 0.0001). SCARA5 is negatively correlated (r = −0.85, *P* = 0.0001) whereas no significant correlation was observed for EIF3K and C1orf123.

### 2.2. Immunohistochemistry

**Figure 2:**
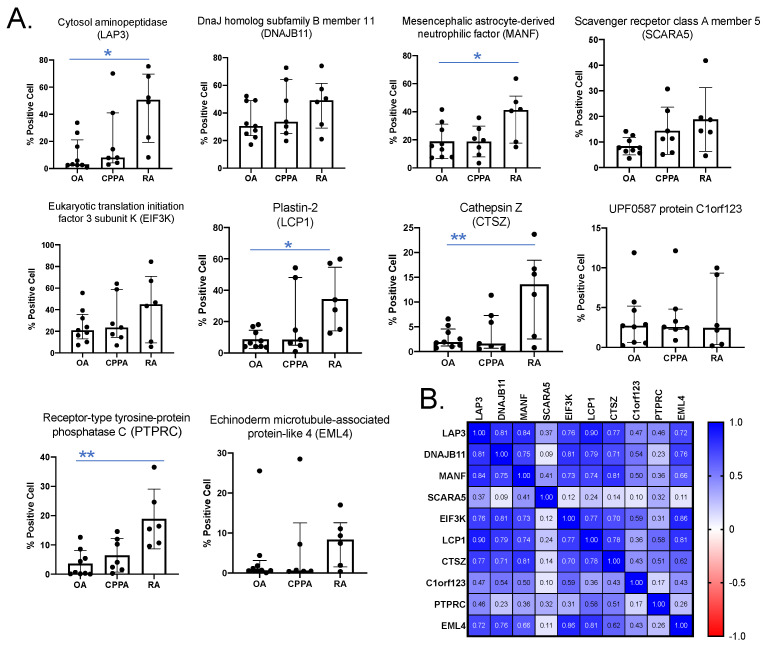
Immunohistochemistry (IHC) quantification of the 10 highlighted proteins in synovial membrane from OA, CPPA and RA patients. (**A**) Representation of protein quantification (optical density values) obtained by QuPath after IHC. One-way ANOVA test (post hoc of Tukey) or Kruskal–Wallis test (post hoc test of Dunn’s) was applied depending on normal distribution: * *P* < 0.05 and ** *P* < 0.01 (**B**) Correlation coefficients between the 10 highlighted biomarkers calculated according to the non-parametric Spearman test. OA, osteoarthritis; CPPA, chronic pyrophosphate arthropathy; RA, rheumatoid arthritis.

Immunohistochemistry was also performed on biopsies for the ten selected biomarkers highlighted by mass spectrometry: LAP3, DNAJB11, MANF, SCARA5, EIF3K, LCP1, CTSZ, C1orf123, PTPRC and EML4. Increased expression levels were statistically significant for five biomarkers (LAP3, MANF, LCP1, CTSZ and PTPRC) in RA biopsies compared to OA (Figure 2A). EML4 values were also increased in RA even though data were not statistically significant (Figure 2A). Correlations between the ten selected proteins intensities obtained by IHC are shown in Figure 2B and the heat map is mostly the same compared to Figure 1C, except for SCARA5, EIF3K and C1orf123.

**Figure 3:**
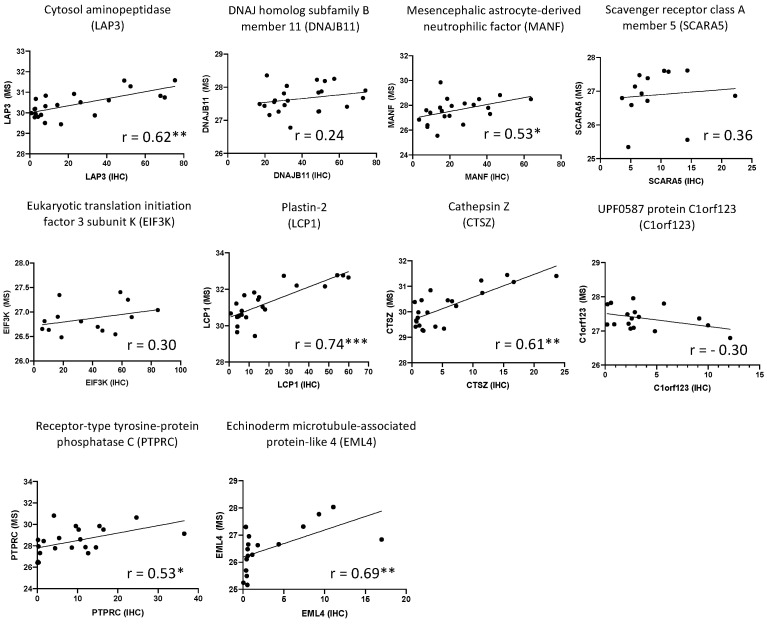
Correlation between mass spectrometry (MS) and immunohistochemistry (IHC) quantification of the 10 highlighted proteins in synovial membrane from OA, CPPA and RA patients. Protein expression levels (Log2 (LFQ)) obtained by mass spectrometry were correlated to the percentage of positive cells obtained by IHC using the non-parametric Spearman test: * *P* < 0.05, ** *P* < 0.01 and *** *P* < 0.001.

Expression levels of LAP3, MANF, LCP1, CTSZ, PTPRC and EML4 obtained by mass spectrometry were all significantly correlated with the corresponding levels obtained by IHC, except for DNAJB11, SCARA5, EIF3K and C1orf123, for which no correlation between both methods was observed (Figure 3).

These biomarkers were also correlated with the histological inflammatory score (Table 1) except for PTPRC: LAP3 (r = 0.83, *P* < 0.0001), MANF (r = 0.70, *P* = 0.0003), LCP1 (r = 0.82, *P* < 0.0001), CTSZ (r = 0.60, *P* < 0.01) and EML4 (r = 0.56, *P* < 0.01). We could not confirm the increase of expression for DNAJB11 in RA biopsies (Figure 2A) but DNAJB11 was correlated with the histological inflammatory score (r = 0.54, *P* < 0.01), suggesting that the most inflamed OA biopsies already presented a high level of DNAJB11 (Table 1). Opposite results were however observed for SCARA5 for which IHC protein levels presented a non-significant trend to increase in the RA group whereas MS/MS proteins levels were significantly decreased in RA (Figure 2A). For IHC analysis, the anti-SCARA5 antibody mainly recognizes a 17 amino acid epitope located at the C-terminal level of the protein. For MS/MS analysis, 13 peptides of SCARA were globally used to quantify the protein, all peptides being not necessarily detected for all biopsies. We observed that the number of peptides used for quantification was distributed for the three groups accordingly (median (range)): 8 (6–12) for OA, 7 (4–10) for CPPA and 3.5 (3–4) for RA. It seems that fewer peptides were used for the quantification of SCARA5 in the RA groups.

The increased levels of EIK3K in CPPA and of C1orf123 in OA detected by MS/MS were not confirmed by IHC (Figure 2A). Finally, all proteins were strongly correlated to each other (*P* < 0.001) except for SCARA5, C1orf123 and PTPRC (Figure 2B).

**Figure 4:**
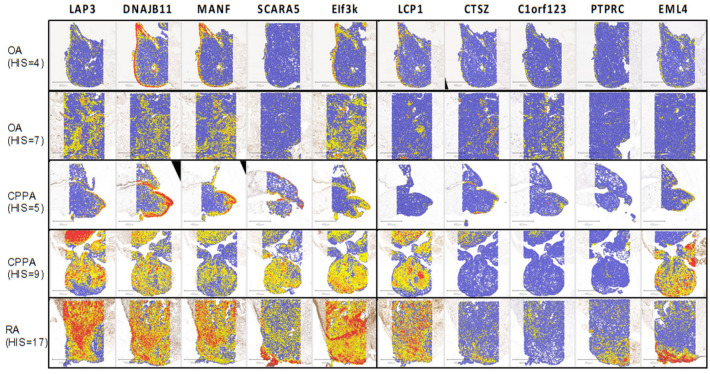
Immunohistochemistry illustration of the 10 highlighted proteins obtained with paraffin-embedded biopsies from synovial membrane of OA, CPPA and RA patients. The histological inflammatory scoring (HIS) is also associated with each biopsy. Highly positive cells have an OD > 0.6 (red spot), moderately positive 0.4 > OD > 0.6 (orange spot), weakly positive 0.2 > OD > 0.4 (yellow spot) and negative cells < 0.2 (blue spot). OA, osteoarthritis; CPPA, chronic pyrophosphate arthropathy; RA, rheumatoid arthritis.

Localization of the 10 proteins by IHC is illustrated on Figure 4 with five biopsies from OA, CPPA or RA patients presenting different histological inflammatory score (HIS).

Negative controls are presented in Appendix A. Distribution of LAP3, DNAJB11, MANF and EIF3K was mainly present in the lining of OA and CPPA with low histological inflammatory score (Figure 4: OA with HIS = 4 and CPPA with HIS = 5), whereas it was strongly present in the stroma of OA, CPPA and RA biopsies with high histological inflammatory score (Figure 4: OA, CPPA and RA with HIS = 7, HIS = 9 and HIS = 17, respectively). SCARA5, LCP1 and EML4 were mainly present in the stroma of CPPA and RA biopsies with high histological inflammatory scores (Figure 4: CPPA and RA with HIS = 9 and HIS = 17, respectively). CTSZ and PTPRC were mainly present in the stroma of RA biopsies. C1orf123 did not present a high percentage of positive cells but was mainly present in the lining of non-inflamed OA biopsies whereas stroma became more positive in inflammatory conditions.

Intensity levels obtained for all ten biomarkers by MS and IHC were correlated to each parameter of the histological inflammatory score (HIS) (Table 1). Considering statistical significance for both MS and IHC analyses, lymphocyte infiltration was significantly correlated with LAP3, MANF, LCP1 and CTSZ; plasmocytes with LAP3 and LCP1; neutrophils with LAP3, MANF, LCP1 and CTSZ and macrophages with LAP3, LCP1, CTSZ and EML4. PTPRC was significantly correlated to lymphocyte, neutrophil and macrophage infiltration by MS/MS and to plasmocyte and neutrophil infiltration by IHC. Hyperplasia was only correlated to MANF and EML4 according to the MS/MS analysis.

Increased or decreased expression obtained by MS and IHC as well as the correlation between MS and IHC, or correlation with the histological inflammatory score (HIS) are summarized in Table 2 for all ten biomarkers.

## 3. Discussion

In our recently published study using the same synovial tissue cohort, we have highlighted an inflammatory continuum at the histological and at the protein level throughout the 24 OA, CPPA and RA synovial membranes [15]. Proteomic analysis of synovial tissue was a powerful tool to identify novel proteins expressed in the synovium of patients with articular disease. In this study, ten protein expressions (LAP3, DNAJB11, MANF, SCARA5, EIF3K, LCP1, CTSZ, C1orf123, PTPRC and EML4) were highlighted by LC-MS/MS as being present in the synovium from RA, but also from OA and CCPA patients; and were significantly increased or decreased in one of the three disease groups.

Except for LCP1 [16,17], CTSZ [16] and PTPRC [18] previously identified in RA and OA synovitis, LAP3, DNAJB11, MANF, SCARA5, EIF3K, C1orf123 and EML4 have never been described in human synovium. This proteomic approach is an additive way to describe and understand the synovial inflammatory continuum described in our previous paper [15]. Of interest, as described later, all these proteins, except EIF3K and C1orf123, exhibit pro- or anti-inflammatory properties and are believed to play a significant role in synovial pannus formation and in immune cells infiltration.

When facing a joint swelling, it is sometimes difficult in daily clinic to distinguish an inflammatory condition such as RA from a degenerative disease such as OA; and a synovial biopsy is a tool that can be used to help the physician to determine a diagnosis (and to choose the proper treatment). Among the ten proteins identified, it should be emphasized that LAP3, DNAJB11, MANF, LCP1, CTSZ, PTPRC and EML4 were significantly increased in RA compared to OA and/or CPPA (with an overexpression confirmed in IHC for LAP3, MANF, LCP1, CTSZ and PTPRC). These seven proteins are therefore overexpressed in RA and their overexpression in the synovial tissue could help to distinguish RA from the two other pathologies. The protein levels of the seven proteins overexpressed in RA were all positively correlated to each other and to the histological inflammatory scoring. Further, when we analyzed the different items of the histological inflammatory score, we mainly observed that there was a correlation between these seven proteins and the different immune cells accumulation (lymphocytes, plasmocytes, polymorphonuclear neutrophils and macrophages), but not with the item “hyperplasia”. It underlines the link between these proteins and the inflammatory component of the infiltration, and not only the synovial hypertrophy.

Cytosol aminopeptidase 3 (LAP3), also called leucine aminopeptidase 3, catalyzes the hydrolysis of leucine residues from the amino termini of protein or peptide substrate. In this study, we observed that LAP3 levels were increased in RA compared to OA and CPPA biopsies and that LAP2 was slightly expressed in the lining border of non-inflamed OA synovial membrane whereas its level of expression is drastically increased in the stroma of OA, CPPA and RA inflamed tissue. LAP3 is strongly correlated with the histological inflammatory score but also to each cell type infiltration. Yang et al. previously observed the presence of LAP3 in colon cancer cells and in surrounding stroma, specifically in lymphocyte infiltrate [19]. LAP3 is described as being involved in the proliferation, migration, invasion and angiogenesis in various cancers such as ovarian, esophageal, breast, colon and liver cancers as well as glioma [20,21,22,23,24]. LAP3 has never been described in OA, CPPA or RA. However, it is strongly correlated with L-plastin-2 or lymphocyte cytosolic protein 1 (LCP1) which is also involved in synovial pannus development. In this proteomic study, we observed the increase of LCP1 expression in RA biopsies compared to OA. LCP1 was mainly expressed in the lining border and punctually in the stroma of the OA synovial membrane whereas its expression considerably increased in the stroma of inflamed synovium. LCP1 is a cytosolic actin-binding protein that belongs to the fimbrin family. It was initially found to be expressed only in hematopoietic cells but recent studies demonstrated that LCP1 also occurs in many nonhematopoietic malignancies such as colon, prostate and breast cancers [25]. LCP1 overexpression contributes to many tumors’ progression and metastasis [26,27]. LCP1 connects the actin cytoskeleton and LFA-1, enabling sustained LFA-1 cluster formation, thus stabilizing the contact between T-cells and APC/target cells through an ICAM-1-LFA-1 interaction [28]. LCP1 supports T-cell activation and motility [29] as well as macrophage motility [30]. In our study, LCP1 was highly correlated with both cell types. Inactivation of LCP1 by antileukoproteinase treatment reduced the frequency and severity of the anti-collagen-II-induced arthritis in mice and has a protective effect against pannus formation and bone erosion [17]. LCP1 phosphorylation regulates the early phase of sealing ring formation by an actin-bundling process in mouse osteoclast [31], whose inhibition leads to their reduced resorptive activities [32].

Another protein predominantly expressed by monocytes, macrophages and dendritic cells, and to a lesser extent by T lymphocytes [33,34] is the Cathepsin Z (CTSZ), also called cathepsin X, which is a lysosomal cysteine protease exhibiting carboxypeptidase activity. In our study, cathepsin Z levels were increased in RA compared to OA biopsies. It was slightly expressed in the lining layer of non-inflamed OA whereas it was further present and even secreted in the stroma of inflamed synovial membranes. Cathepsin Z is expressed in prostate and gastric carcinoma as well as in macrophages of gastric mucosa, especially after infection with Helicobacter pylori infection, but also in glial cells [33]. Through activation of β2 integrin receptor Mac-1 (CD11b/CD18), cathepsin Z enhances the adhesion of monocytes/macrophages to fibrinogen as well as maturation of dendritic cells, a process crucial in the initiation of adaptive immunity [33]. Through activation of the other β2 integrin receptor LFA-1 (CD11a/CD18), cathepsin Z is involved in the proliferation and migration of T lymphocytes [33,34]. Lastly, procathepsin Z has an RGD domain that binds to αvβ3 integrin therefore modulating the attachment of migrating cells to ECM component [35]. Further macrophage-secreted cathepsin Z facilitates cancer cell invasion through RGD-dependent binding of integrin receptor [36]. The pathophysiological role of cathepsin Z was recently reviewed [37] as well as its extracellular role [38]. Cathepsin Z has not yet been extensively described in OA, CPPA or RA.

Our proteomic study also highlighted two anti-inflammatory proteins, scavenger receptor class A member 5 (SCARA5) and mesencephalic astrocyte-derived neurotrophic factor (MANF). SCARA5 is a member of the class A scavenger receptors. It contributes to the clearance of pro-inflammatory molecules (e.g., HMGB1 molecule), foreign particles and pathogens [39,40]. In mice, SCARA5 is found in a subset of fibroblast-like cells (positive for PDGFRα and vimentin) in the interstitial stroma of most organs, with additional expression in the epithelial cells of testis and choroid plexus [41]. In our study, IHC also highlighted the presence of SCARA5 in the lining of non-inflamed synovial membrane, which is coherent with the clearance of pro-inflammatory molecules inside the synovial fluid by the synovial membrane lining layer. SCARA5 was also expressed in the stroma of CPPA and RA biopsies, which contradicts the decrease of expression observed by mass spectrometry. This controversial result could be related to the targeted sequence for quantification by both approaches. SCARA5 has been described as a tumor suppressor in various cancers [42,43,44,45,46]. SCARA5 knockdown markedly enhances tumor growth, invasiveness and metastasis. Conversely, overexpression of SCARA5 inhibits tumor proliferation and invasion [44]. Outside cancer, SCARA5 is also a positive regulator in adipocyte lineage commitment and differentiation [47]. Knockdown of SCARA5 inhibits human aortic smooth muscle cell proliferation and migration, a critical step in the progression of atherosclerosis [48]. SCARA5-null mice develop with age lymphoid cell accumulation in many organs (lungs, skin, liver and adipose tissue) and show decreased endocytic function in fibroblasts. Furthermore, about one-third of the mice develop antinuclear antibodies. These disturbances are reminiscent of those found in many human autoimmune connective tissue disorders (Sjogren’s syndrome, lymphocytic interstitial pneumonia), which suggests that defects in fibroblast SCARA5 can underlie some forms of autoimmune diseases [41]. MANF was initially discovered as an astrocyte-derived factor but it was also recently detected in immune cells [49]. It is localized in the endoplasmic reticulum, whose expression and secretion are increased under ER stress [50] or pro-inflammatory cytokines [51]. MANF mRNA is highly increased in peripheral white blood cells of RA patients as well as in the synovium of rabbit arthritis model, for which MANF was mainly localized in the cytoplasm of a-SMA-positive FLS and poorly in CD68-positive macrophage-like synoviocytes [50]. In our study, we observed that MANF levels were increased in RA compared to OA and that it was highly expressed in inflamed synovium. MANF was also highly correlated with the lymphocyte and plasmocyte but not with the macrophage score. Chen et al. observed that MANF could suppress the expressions of NF-kB-dependent target genes and the proliferation of inflammatory synoviocytes [50]. MANF also promotes immune cell phenotype switch from proinflammatory macrophages to pro-repair anti-inflammatory macrophages [49]. MANF is therefore considered as a negative regulator of inflammation [52]. Further, restoring MANF levels can extend fly lifespan, reverse liver damage and inflammation in old mice by regulating metabolic and immune homeostasis in ageing [49,53]. The cytoprotective and immune-modulatory functions of MANF are likely to synergize for promoting tissue recovery [54]. These recent studies highlight the therapeutic application for MANF in inflammatory diseases. ERdj3 (DNAJB11) is another ER stress protein that acts as a co-chaperone of BiP enhancing its ATPase activity [55]. It was also identified as a secreted chaperone preventing misfolded protein aggregation by accompanying them extracellularly to reduce their proteotoxic effect [56]. Its expression is correlated with the histological inflammatory score and is highly increased in the stroma of inflamed synovium among the three groups. However, its role needs to be better defined in synovitis.

As leukocyte infiltration is one of the hallmarks of RA synovitis, it was expected to observe by MS/MS and IHC an increase of expression of PTPRC, also called leukocyte common antigen or CD45. The protein is expressed on all nucleated hematopoietic cells and their precursors, except mature erythrocytes and platelets and functions as a key regulator of T and B cell signaling [57]. CD45 is expressed in several isoforms that depend on the stage of immune cells maturation, activation and differentiation. It represents 5–10% of the total glycoprotein on the surface of T- and B-lymphocytes [57]. PTPRC was significantly correlated to leukocyte infiltration according to the IHC or MS/MS analysis.

Echinoderm microtubule-associated protein-like 4 (EML4) is involved in cancers when spliced with the anaplastic lymphoma kinase. The reason is an exchange of chromosomal segments on the short arm on chromosome 2 (2p23) leading to the formation of chimeric EML4-ALK fusion oncoprotein, which possesses potential oncogenic functions due to its constitutive activation of ALK kinase [58]. EML4-ALK oncoprotein is associated with 6.7% of the non-small-cell lung cancer [59] and 2% of medullary thyroid cancer [60]. EML4 was mainly expressed in the lining border and punctually in the stroma of OA synovial membranes, whereas its expression considerably increased in the stroma of highly inflamed synovium. EML4 was correlated with the histological inflammatory scoring and specifically with macrophage and lymphocyte infiltration (by MS/MS only).

Lastly, Eukaryotic translation initiation factor 3 subunit K (EIF3K) and C1orf123 (chromosome 1 open reading frame 23) levels were increased by MS/MS in CPPA and OA, respectively, compared to the two other groups. However, it was not confirmed by IHC. EIF3K was detected on the lining of non-inflamed synovium and in the stroma in inflammatory conditions as also observed for C1orf123 but to a lesser extent. They were not correlated to the histological inflammatory scoring or to any related independent parameters. EIF3K is the smallest subunit of the eIF3 complex, which controls the regulation of gene expression and the initiation of protein synthesis. Aberrant expression of various eIF3 subunits were detected in various human cancers [61] but not for eIF3K. It remains therefore unknown whether eIF3K contributes or regulates the activity of the eIF3 complex in translational initiation in vivo [62]. C1orf123, also known as UPF0587 protein, was identified in goats as an adipokine that may be involved in endocrine functions [63]. Human C1orf123 is proposed as one of the human O-GlcNAc transferase interactors, playing a potential role in post-translation modification [64]. C1orf123 was found to be a novel zinc-binding protein [65,66] that could play a role in mitochondrial oxidative phosphorylation [66] and could interact specifically with the heavy metal-associated domain of a copper chaperone for superoxide dismutase [65].

We therefore detected the increase of expression of seven proteins (LAP3, DNAJB11, MANF, LCP1, CTSZ, PTPRC and EML4) in RA biopsies that was confirmed by IHC except for DNAJB11 for which levels of expression were already elevated in inflamed OA biopsies. Protein levels all correlated to the histological inflammatory score suggesting that they contribute to cell proliferation and/or leukocyte infiltration, except MANF that is described as a mediator in the inflammation resolution. Immunohistological experiments highlighted the presence of these proteins mainly in the stroma of inflamed RA biopsies but also in some inflamed OA and CPPA biopsies for LAP3, DNAJB11 and MANF. The latter also presented a high level on the lining of non-inflamed biopsies suggesting another function in physiological condition and their expression by other cell types.

Discrepancies between MS and IHC data sometimes occur. Protein detection by immunohistochemistry is dependent on epitope accessibility and its affinity for the antibody. Detection of proteins by MS is also dependent on peptide ionization and their ability to escape ion suppression. Therefore, it is a real success once protein expression can be correlated by both approaches. When opposite results appear, other experiments are requested. In our study, the expression of SCARA5 was controversial due to the decreased levels detected in RA biopsies by mass spectrometry, whereas the increased levels were observed in the stroma of inflamed biopsies by immunohistochemistry. This should be clarified in future work, suggesting that both approaches (MS/MS and IHC) do not target the same SCARA5 peptides generating therefore opposite effect. We do not exclude that the protein might be truncated in the inflammatory condition or that some peptides might be post-translationally modified. Further, it should be emphasized that MS analysis is performed on proteins extracted from biopsies (presenting a three-dimensional structure) whereas IHC is performed on a small slice of the biopsy (presenting a two-dimensional structure). However, studying SCARA5 remains of interest as it is described as a negative regulator of cell proliferation and invasion. Finally, the increase of expression of eIF3K in CPPA and of C1orf123 in OA has not been confirmed by immunohistochemistry and was not correlated to inflammatory parameters. Nevertheless, eIF3K seems to be highly expressed in the lining of non-inflamed biopsies, whereas its expression is further located in the stroma in inflammatory conditions. The role played by all these proteins in the pannus formation is yet to be described in the arthritis synovitis. Therefore, they request further attention, especially in the assessment of their expression patterns in various cell types. Our cohort is a cohort of investigation including a relatively low number of samples dedicated to high-throughput proteomic analysis. In this study, we decided to keep intra-group heterogeneity in regard to what is encountered in daily clinical routine. Among the three pathologies, RA is probably the most heterogenous one. RA can be classified, inter alia, into four major phenotypes lymphoid, myeloid, low inflammatory, and fibroid presenting different response to therapy [67]. We also observe seronegative and seropositive RA patients in our cohort. Seropositive RA (FR+ and/or ACPA+) may represent a subset with several distinguishing features, in regard to seronegative (FR-, ACPA-) RA. Floudas et al. identified that CD4 T cell proinflammatory cytokine production was markedly different between ACPA− and APCA+ RA patients, even though there was no difference with the B cells signature [68]. It is also known that ACPA+ RA patients have a higher radiological severity score with more erosion [69]. New studies including more patients classified into different subgroups, are now requested to clarify whether the expression of our proteins of interest can be associated to one of these phenotypes. Presence of infiltrated immune cells certainly contribute to the expression or secretion of these proteins. Colocalization of these proteins with immune cells by the classically used immunohistochemistry approach or by a yet to be technology such as single-cell proteomics would be of high interest.

## 4. Materials and Methods

### 4.1. Patients and Synovial Tissue

All experiments undertaken with patient material complied with the regulations and ethical guidelines of the CHU of Liege, Belgium and were approved by the CHU ethical committee (B707201732662; ref: 2017/147). Informed consent was obtained from all subjects. Patients with knee OA (n = 9), CPPA (n = 7) and RA (n = 8) were selected from a retrospective cohort of 137 patients with arthritis diseases, according to clinical examination, serological analysis and histological inflammatory scoring of synovial membranes. Synovial biopsies were obtained from each patient by needle arthroscopy as described previously [15] and stored at −80 °C until used for proteomic studies. Other fragments were also embedded in paraffin for the histological inflammatory scoring and the immunohistochemistry (IHC). Clinical and biological data were previously summarized [15]. Briefly, parameters related to age (median: 55, 65 and 57 years), gender (% of woman: 88, 71 and 62) and BMI (median: 32, 24, 24 kg/m^2^) were not statistically different between OA, CPPA and RA patients, respectively.

Kellgren and Lawrence grade (K&L) [70] defined the disease severity of OA (median (min–max): 3 (0–4)) and CPPA (median (min–max): 2 (0–4)) and was not statistically different between the two groups. The histological inflammatory scoring was previously described [15] and based on Tak’s score [71]. Briefly, the histological inflammatory scoring was scored using hematoxylin eosin-stained section of synovial biopsies and included the sum of the following components: synovial hyperplasia (hy; 0–4 score) and the degree of infiltration of lymphocytes (ly; 0–4 score), plasma cells (pl; 0–4 score), polymorphonuclear cells (PMN; 0–3 score). Macrophage infiltration was also included based on CD68 expression (0–3 score) obtained by immunohistochemistry. Accordingly, the histological inflammatory score was set as the sum of the previously cited components leading to a maximum of 18. The histological inflammatory score obtained for each disease group was the following: median (min–max) of 4 (3–8) for OA, 5 (5–13) for CPPA and 14 (12–17) for RA. The histological inflammatory score was significantly different between the three groups (*P* = 0.0003) and was higher for RA compared to OA (*P* < 0.001) or CPPA (*P* < 0.05), but not different between OA and CPPA groups. CRP values exceeding the normal range were observed in 20%, 40% and 90% of OA, CPPA and RA patients, respectively. RA patients were positive for the rheumatoid factor, the anti-citrullinated protein antibodies (ACPA) and the erythrocyte sedimentation rate (ESR) at 40%, 60% and 60%, respectively. All patients were untreated by corticosteroids or any disease-modifying antirheumatic drugs (DMARDs), including biologics, at the time of sampling.

### 4.2. D-nano-UPLC-ESI-Q-Orbitrap for Proteomic Analysis

The method has been extensively described elsewhere [15]. Briefly, 5 mg of the synovial biopsy were resuspended in RIPA buffer and disrupted to allow proteins solubilization in RIPA buffer. Proteins (15 µg) at a concentration of 0.5 μg/μL in 50 mM ammonium bicarbonate were then reduced (DTT), alkylated with iodoacetamide and precipitated using the 2D clean-up kit (GE Healthcare, Diegem, Belgium). Protein pellets were digested with trypsin, and 3.5 μg of peptides were desalted using Ziptip C18 (Millipore Corp., Billerica, MA, USA). Before injection into the 2D-nano-UPLC system, the digested proteins were resuspended at a concentration of 2.5 µg in 9 μL of 100 mM ammonium formate solution adjusted to pH 10. Each sample was then spiked with a standard MassPREP digestion mixture (MPDS Mix) (Waters Corp., Milford, MA, USA) at a quantity of 150 fmoles of ADH digest per injection. All samples were injected on a 2D-nanoAquity UPLC (Waters, Corp., Milford, MA, USA) coupled online with an ESI-Q-Orbitrap (Q Exactive, Thermo Fisher Scientific, Waltham, MA, USA) in positive ion mode, as previously described [72]. Briefly, for the 2-dimensional method (2D-LC), three steps were applied on a high pH column with an increasing percentage of acetonitrile. Eluted peptides were then injected on a low pH column using a gradient from 99% to 93% of buffer A (A = H2O, 0.1% formic acid, B = acetonitrile, 0.1% formic acid) for 5 min, followed by a gradient from 93% of A to 65% of A during 135 min (total run time of 180 min per fraction). TopN-MS/MS was used for the acquisition method selecting the 12 most intense peaks on the Full MS spectrum (singly charged precursors excluded). Full MS2 spectrum was then acquired for these 12 compounds. MS acquisition parameters were the following: mass range from 400 to 1750 m/z, resolution of 70,000, automated gain control (AGC) target of 106 or maximum injection time of 200 ms. MS2 acquisition parameters were: isolation window of 2.0 m/z, collision energy (NCE) of 25, resolution of 17,500, AGC target of 105 or maximum injection time of 50 ms. Proteins were identified by the software MaxQuant ver.1.5.2.8. The false discovery rate (FDR) both at the Peptide Spectrum Match (PSM) and at the protein levels was set at 0.01 (1%) in MaxQuant.

### 4.3. Immunohistochemistry

Immunohistochemistry was performed on 22 out of the 24 biopsies, 2 RA biopsies being unavailable for IHC. Biopsies were fixed in 4% paraformaldehyde for 24h at 4 °C, dipped in 70% (*v*/*v*) ethanol and then embedded in paraffin. For IHC, slides were first heated overnight at 65 °C. The day after, sections were dewaxed in xylene and subsequently passed through 100% ethanol and 70% ethanol. Antigen retrieval was then performed with a steamer for 10 min in a target retrieval solution (Agilent, Santa Clara, CA, USA). Endogenous peroxidases were blocked with 3% H2O2 for 20 min followed by blocking with Dako-Real antibody diluent (Agilent). Sections were incubated (or not for negative controls) O/N with a primary antibody against LAP3 (Abcam, #ab154809, dil 1/100), DNAJB11 (Abcam, #ab224082, dil 1/200), MANF (Abcam, #ab67271, dil 1/400), SCARA5 (Abcam, #ab118894, dil 1/200), eIF3k (Thermofisher, #PA5-27593, dil 1/1000), LCP1 (Cell signaling, #3588S, dil 1/400), CTSZ (Abcam, #ab204303, dil 1/100), C1orf123 (Abcam, #ab122865, dil 1/25), PTPRC (Abcam, ab10558, dil 1/500) or EML4 (Cell signaling, #12548, dil 1/1000). Rinsed slides were incubated with EnVision + System-HRP labeled polymer anti-rabbit (Agilent) in a humidified chamber for 30 min at RT. Peroxidase was revealed with a Liquid DAB+ Substrate Chromogen System (Agilent) and sections were counterstained with Carazzy’s hematoxylin (EMD Millipore, Billerica, MA, USA). Staining was revealed with Nanozoomer Digital Pathology 2.0 HT scanner (Hamamatsu photonics, Hamamatsu, Japan) and quantified using QuPath software [73]. Hematoxylin-eosin staining was performed according to classical protocols.

### 4.4. Quantification of IHC Using QuPath

QuPath version v0.2.3 was downloaded from Github (https://QuPath.github.io/, last accessed date 14 November 2021) [73]. A new project was created on QuPath for each protein examined. This step was performed to avoid resetting all parameters for each image related to the same antibody. Concerning calibration of DAB intensity, the image type was set to H-DAB, DAB being the chromogen used. RGB values for DAB were calibrated by selecting a representative region of the background and stained tissue.

The default settings were used for sample tissue detection except for thresholds that were adapted for tissue recognition. Then, artifacts were eliminated manually to avoid false positives. Default settings were also used for positive cell detection and quantification. For quantification, cells were considered as positive to the DAB staining when the optical density (OD) was > 0.2. For image representation, highly positive cells had an OD > 0.6 (red spot), moderately positive 0.4 > OD > 0.6 (orange spot), weakly positive 0.2 > OD > 0.4 (yellow spot) and negative cells < 0.2 (blue spot).

### 4.5. Data Analysis

For mass spectrometry analysis: Protein identification (based on MS/MS spectra) and protein quantification (based on MS1 intensities) were performed by Maxquant analysis. For quantification, proteins intensities were normalized using the LFQ algorithm in Maxquant [74], imported in Perseus software (version 1.5.5.0) and Log2 transformed for comparison between samples. In total, 1871 proteins were selected for statistical analysis based on their quantification in at least 7 biopsies of at least one of the three disease groups (OA, CPPA and/or RA). The multiple sample test (1400 permutation-based FDR, FDR set at 0.05) was used to compare the three group’s protein intensities [OA, CPPA and RA]. One-way (ANOVA) with a post hoc test of Tukey was then applied on the 10 selected biomarkers after verifying that all values passed the D’Agostino normality test. Correlation coefficients (between the 10 selected biomarkers and versus the histological inflammatory score) were obtained using the Pearson test.

For IHC: D’Agostino normality test was used to assess values distribution. A one-way ANOVA test followed by Tukey post hoc test for multiple comparisons was applied for values that passed the normality test. For values that did not pass the normality test, the Kruskal-Wallis test followed by Dunn’s post hoc test for multiple comparisons was applied. Correlation coefficients (among the 10 selected biomarkers but also versus the histological inflammatory score) were obtained using the Spearman test as all values did not pass the D’Agostino normality test. Correlations between mass spectrometry data and IHC were performed by using the non-parametric Spearman test.

## Figures and Tables

**Table 1:** Correlation between quantified proteins and each parameter of the histological inflammatory scoring (HIS). Mass spectrometry: correlation parameters calculated by correlating MS-Log2 protein intensities and HIS parameters. Immunohistochemistry: correlation parameters calculated by correlating IHC-protein intensities and HIS parameters. MS, mass spectrometry; IHC, immunohistochemistry; HIS, histological inflammatory scoring; hs, hyperplasia; ly, lymphocyte; pl, plasmocyte; PMN, polymorphonuclear neutrophils; MΦ, macrophage. Methods used for quantifying protein intensities are in bold print. ** *P* < 0.01 and *** *P* < 0.001.

Mass Spectrometry												
MS	HIS (0–18)		hs (0–4)		ly (0–4)		pl (0–4)		PMN (0–3)		MΦ (0–3)	
LAP3	0.77	***	0.38		0.78	***	0.67	***	0.63	***	0.61	**
DNAJB11	0.77	***	0.43		0.71	***	0.81	***	0.58	**	0.48	
MANF	0.79	***	0.59	**	0.75	***	0.80	***	0.54	**	0.41	
SCARA5	−0.85	***	−0.33		−0.90	***	−0.62		−0.80	***	−0.65	**
EIF3K	0.19		0.13		0.33		−0.03		0.06		0.37	
LCP1	0.74	***	0.30		0.73	***	0.59	**	0.61	**	0.74	***
CTSZ	0.69	***	0.39		0.62	**	0.54	**	0.63	**	0.63	**
C1orf123	−0.37		−0.36		−0.34		−0.25		-0.16		−0.42	
PTPRC	0.68	***	0.22		0.68	***	0.52		0.54	**	0.75	***
EML4	0.78	***	0.58	**	0.76	***	0.66	**	0.55		0.67	**
**Immunohistochemistry**												
**IHC**	**HIS (0–18)**		**hs (0–4)**		**ly (0–4)**		**pl (0–4)**		**PMN (0–3)**		**M** **Φ** **(0–3)**	
LAP3	0.83	***	0.48		0.78	***	0.62	**	0.62	**	0.68	***
DNAJB11	0.54	**	0.30		0.49		0.33		0.32		0.45	
MANF	0.70	***	0.50		0.68	***	0.52		0.55	**	0.51	
SCARA5	0.40		0.30		0.46		0.47		0.22		0.20	
EIF3K	0.47		0.21		0.41		0.34		0.46		0.47	
LCP1	0.82	***	0.48		0.71	***	0.70	***	0.63	**	0.69	***
CTSZ	0.60	**	0.38		0.57	**	0.39		0.64	**	0.55	**
C1orf123	0.07		−0.17		0.02		0.10		0.02		−0.05	
PTPRC	0.48		0.23		0.40		0.58	**	0.67	***	0.41	
EML4	0.56	**	0.31		0.48		0.39		0.31		0.56	**

**Table 2:** Overview table related to the 10 highlighted proteins. Summary concerning modulated protein expression detected by mass spectrometry (MS/MS) or immunohistochemistry (IHC) and correlated to the histological inflammatory scoring (HIS). “V” means detected by the method or correlated; “X” means not detected by the method or not correlated. * Not significant.

		Method	Method	Correlation	Correlation	Correlation	Localization by IHC
		MS/MS	IHC	MS/MS vs IHC	MS/MS vs HIS	IHC vs HIS	
Increased expression in RA	LAP3	V	V	V	V	V	Lining in OA/CPPA with low HIS; Stroma of OA/CPPA/RA with high HIS
	DNAJB11	V	X	X	V	V	Lining in OA/CPPA with low HIS; Stroma of OA/CPPA/RA with high HIS
	MANF	V	V	V	V	V	Lining in OA/CPPA with low HIS; Stroma of OA/CPPA/RA with high HIS
	LCP1	V	V	V	V	V	Stroma of CPPA/RA with high HIS
	CTSZ	V	V	V	V	V	Stroma of RA with high HIS
	PTPRC	V	V	V	V	X	Stroma of RA with high HIS
	EML4	V	(V) *	V	V	V	Stroma of CPPA/RA with high HIS
Decreased expression in RA	SCARA5	V	X	X	V	X	Stroma of CPPA/RA with high HIS
Increased expression in CPPA	EIF3K	V	X	X	X	X	Lining in OA/CPPA with low HIS; Stroma of OA/CPPA/RA with high HIS
Increased expression in OA	C1orf123	V	X	X	X	X	Not a high percentage of positive cells

**Figure A1:**
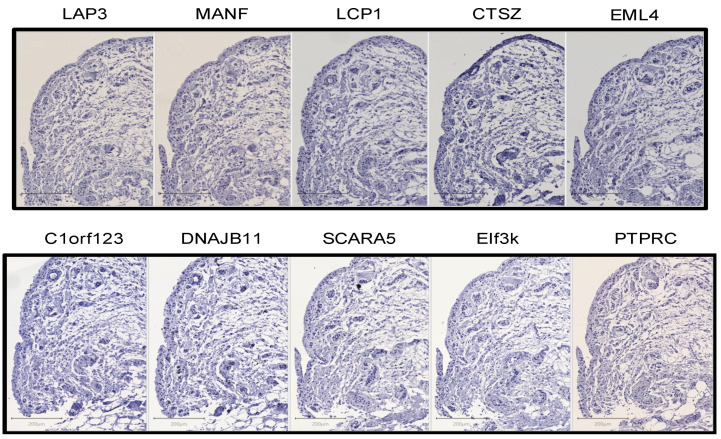
Immunohistochemistry Illustration of Negative Controls Obtained for the 10 Highlighted Proteins.

## Data Availability

The datasets used and/or analyzed during the current study are available from the corresponding author on reasonable request.
